# The Transcriptional Repressor, MtrR, of the *mtrCDE* Efflux Pump Operon of *Neisseria gonorrhoeae* Can Also Serve as an Activator of “off Target” Gene (*glnE*) Expression

**DOI:** 10.3390/antibiotics4020188

**Published:** 2015-06-03

**Authors:** Paul J. T. Johnson, William M. Shafer

**Affiliations:** 1 Department of Microbiology and Immunology, Emory University School of Medicine, Atlanta, GA 30322, USA; E-Mail: Paul.Johnson@ung.edu; 2 The Emory Antibiotic Resistance Center, Emory University School of Medicine, Atlanta, GA 30322, USA; 3 Laboratories of Bacterial Pathogenesis, VA Medical Center, Decatur, GA 30033, USA

**Keywords:** drug efflux, DNA-binding protein, glutamine biosynthesis, *Neisseria gonorrhoeae*, transcriptional control

## Abstract

MtrR is a well-characterized repressor of the *Neisseria gonorrhoeae* *mtrCDE* efflux pump operon. However, results from a previous transcriptional profiling study suggested that MtrR also represses or activates expression of at least sixty genes outside of the *mtr* locus. Evidence that MtrR can directly repress so-called “off target” genes has previously been reported; in particular, MtrR was shown to directly repress *glnA*, which encodes glutamine synthetase. In contrast, evidence for the ability of MtrR to directly activate expression of gonococcal genes has been lacking; herein, we provide such evidence. We now report that MtrR has the ability to directly activate expression of *glnE*, which encodes the dual functional adenyltransferase/deadenylase enzyme GlnE that modifies GlnA resulting in regulation of its role in glutamine biosynthesis. With its capacity to repress expression of *glnA*, the results presented herein emphasize the diverse and often opposing regulatory properties of MtrR that likely contributes to the overall physiology and metabolism of *N. gonorrhoeae*.

## 1. Introduction

DNA-binding proteins that regulate expression of efflux pump-encoding genes have significance in controlling levels of bacterial resistance to clinically important antibiotics and other antimicrobials [1,2,3,4,5]. A well-established, and often repeated, observation is that mutations that abrogate the DNA-binding ability of these regulators or expression of their respective gene can, depending on the nature of the protein, increase or decrease efflux pump gene expression and levels of bacterial resistance to antimicrobials recognized by the cognate efflux pump [6,7,8,9,10,11,12]. Results from recent studies suggest, however, that such DNA-binding proteins also participate in the regulation of other genes that are important in the overall biology of the bacterium [7,13,14]; we have termed such regulated genes as being “off-target” [14].

**Figure 1:**
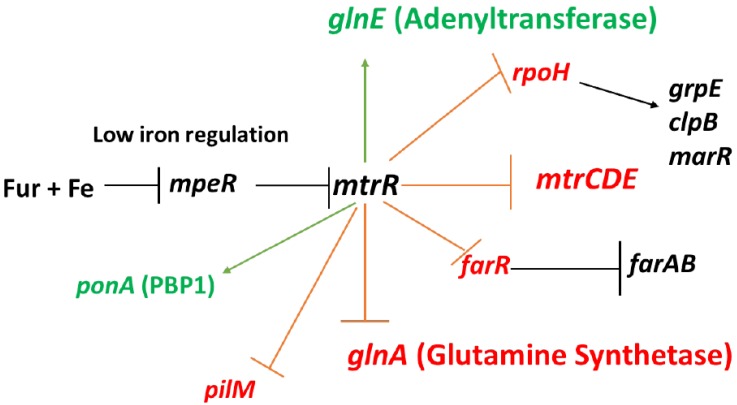
MtrR regulation of gonococcal genes. Shown are genes subject to direct or indirect regulation by MtrR (see text for details). Genes under negative control by MtrR are indicated by a red barred line extending from *mtrR* while those activated by MtrR are indicated by a green arrow extending from *mtrR*; other important regulated genes are shown with black arrowed or barred lines to signify transcriptional activation or repression of expression, respectively. Repression of *mtrR* expression by the product of the *mpeR* gene which is in-turn negatively controlled by Fur + iron are also shown.

While a considerable amount of information is available regarding how DNA-binding proteins control efflux pump gene expression, less is known about how they influence the expression of “off-target” genes and the impact such regulation has for bacterial physiology and pathogenesis. For instance, results from a transcriptional profiling study performed by us [7] defined the MtrR regulon of *N. gonorrhoeae* strain FA19 as consisting of at least 47 repressed and 22 activated genes. MtrR is a member of the TetR/QacR family of DNA-binding proteins that are known for their capacity to repress bacterial genes encoding drug efflux proteins [2,8,15,16]. MtrR represses transcription of the *mtrCDE* efflux pump operon [15,16] by binding as two homodimers to a DNA sequence containing two pseudo-direct repeats within the *mtrCDE* promoter region [8,10]. Missense mutations that cause radical amino changes (e.g., A39T or G45D) in the helix-turn-helix motif of MtrR significantly decrease its DNA-binding activity resulting in increased expression of *mtrCDE* and decreased susceptibility of gonococci to hydrophobic drugs, dyes, and detergents [11]. We hypothesize that such regulation (and loss thereof) is important *in vivo*, as null mutations in *mtrR* increase fitness of gonococci *in vivo* when assessed in a female mouse model of lower genital tract infection [17,18]. Expression of *mtrR* is controlled by a *cis*-acting 13 bp inverted repeat sequence within the *mtrR* promoter, which overlaps the adjacent but divergent *mtrCDE* promoter; a single bp deletion within this sequence abolishes *mtrR* expression [15,19]. Expression of *mtrR* is also subject to repression by the product of the *mpeR* gene [20], which is in-turn repressed by Fur (Ferric Uptake Regulator) in the presence of iron [21] (Figure 1).

In addition to repressing expression of *mtrCDE*, MtrR also directly down-regulates expression of other genes such as: *farR* (encodes the repressor of the *farAB* efflux pump operon) [9], *rpoH* (encodes the major stress-related sigma factor produced by gonococci) [7], *ponA* (encodes penicillin-binding protein 1) [13], and *glnA* (encoding glutamine synthetase) [7,14]; these regulatory schemes are summarized in Figure 1. MtrR also indirectly activates gonococcal genes such as *pilM* [7] and *farAB* [9], but evidence for its capacity to directly activate genes has heretofore been lacking. In order to test if MtrR can serve as a direct activator of gonococcal genes, we studied its regulation of *glnE* since its expression was significantly decreased in an MtrR-negative mutant compared to the wild-type parent strain [7].

We hypothesize that the fidelity of the glutamine biosynthesis pathway is important for optimal physiology and metabolism of gonococci during infection since the levels of this amino acid are very low in mucosal fluids and within phagocytes compared to the levels found in blood [22]. Further, since *glnE* has been reported to be an essential gene in other pathogens such as *Mycobacterium tuberculosis* [23,24] and we have been unable to construct a gonococcal *glnE* null mutant (data not presented), we posit that the opposing regulatory properties of MtrR on *glnA* and *glnE* likely serves to modulate the fidelity of glutamine biosynthesis during infection.

## 2. Results and Discussion

Given our inability (see above) to isolate a *glnE* null mutant of gonococci (data not presented), which suggests that this gene is essential gene in this pathogen), and the results from transcriptional profiling studies that suggested it is an MtrR-activated gene [7], we hypothesize that MtrR regulation of *glnE* is of importance for fidelity of glutamine biosynthesis and the overall physiology of gonococci. Accordingly, to test this possibility we examined if such regulation is direct or indirect and used DNA-binding assays to distinguish these possibilities. This is an important regulatory issue to resolve as MtrR can indirectly activate other genes (e.g., the *farAB* efflux operon) by its ability to repress expression of genes encoding other transcriptional repressors (e.g., *farR*) [9]. In contrast to its previously reported ability to repress the expression of *mtrCDE* [8,10,11] and other gonococcal genes [7,12,13,14], microarray work by Folster *et al.* [7] indicated that MtrR significantly (seven-fold; *p* ≤ 0.05) enhances *glnE* expression in strain FA19. In confirmation of this report, using qRT-PCR with RNA extracted from MtrR-positive strain FA19 and its MtrR-negative deletion mutant strain JF1 [7,17], we found a nine-fold reduction in the *glnE* transcript level in strain JF1 compared to parental strain FA19 (data not presented).

**Figure 2:**
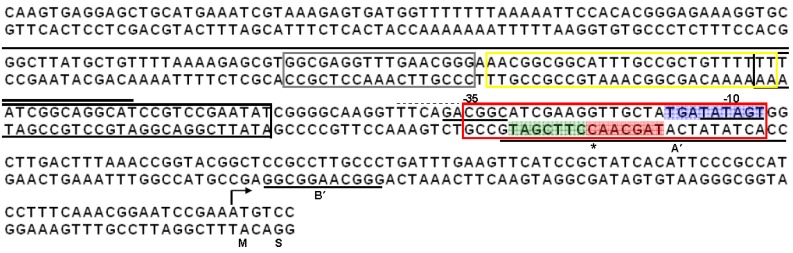
The nucleotide sequence upstream of *glnE* and MtrR-binding sites. The 301 bp sequence of the DNA upstream of *glnE* and the first two codons (encoding M and S, respectively) is shown with the annotated −10 and −35 hexamer sequences of the *glnE* promoter identified by a line under the sequences. The putative extended −10 element is shown in blue. The alternative −35 hexamer is shown by the dashed line above the sequence. The boxed regions represent predicted MtrR binding sites that were identified based on sequence similarity to that of a site upstream of *mtrCDE* [8,10] or *rpoH* [7]. The grey box represents a sequence with 53% identity to the region upstream of *rpoH* [7] while the yellow, black, and red boxes represent sequences with 55%, 67%, and 52%, respectively, identity to regions upstream of *mtrCDE* [8,10]. The MtrR-binding sites identified by DNase I protection (Figure 3) are noted by the solid line above the sense strand or below the anti-sense strand; the two sites on the anti-sense strand are denoted as A′ and B′ with the DNase I hypersensitive site in A′ shown by an *. The adjacent seven nucleotide imperfect inverted element is shown in green and red.

**Figure 3:**
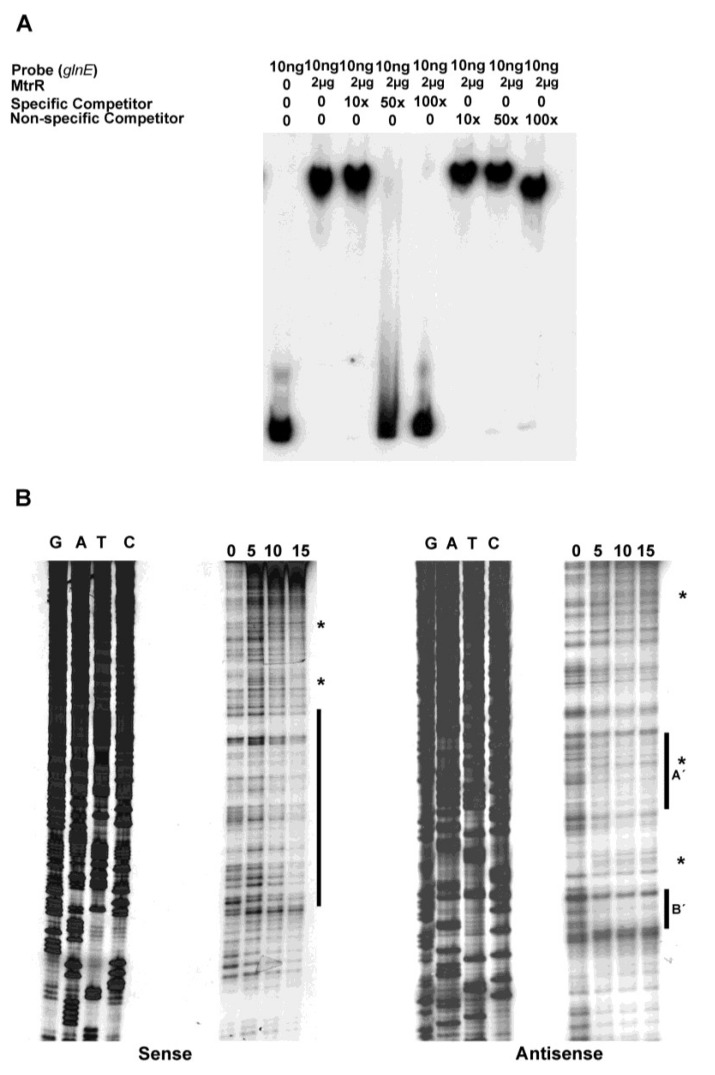
Identification of the MtrR-binding site in the *glnE* upstream DNA. (**A**) The binding specificity of MtrR for the DNA shown in Figure 1 was determined by competitive EMSA; (**B**) The MtrR-binding sites within this sequence were identified by DNase I protection assays that employed increasing amounts of purified MtrR-MBP (0, 5, 10, and 15 μg) with both sense and anti-sense probes. The protected regions on each probe are identified by the black bars and the two sites on the anti-sense strand are labeled as A′ and B′. Regions containing DNase I hypersensitive sites, which could contain more than one nucleotide, on the sense and antisense strands are denoted by *. The sequencing reactions for each probe are adjacent to the DNase I protection reactions and oriented G, A, T, C.

In order to determine if MtrR activation of *glnE* is by a direct mechanism, we tested if it can bind to the sequence upstream of *glnE* (Figure 2). Based on the published MtrR-binding site sequences upstream of *mtrCDE* [7,10], *farR* [9], and *rpoH* [7], we detected four potential MtrR-binding sites on the sense and anti-sense strands in this sequence, which ranged in sequence identity from 67% to 52% for regions on the sense and anti-sense strands, respectively. Using competitive EMSA, we confirmed that the DNA sequence upstream of *glnE* could bind MtrR (Figure 3A) in a specific manner as such binding was competed by unlabeled specific DNA. The nonspecific DNA failed to show such competition, although at its highest level (100× excess) the electrophoretic mobility of the MtrR::*glnE* DNA complex was slightly faster than at lower concentrations of competing DNA; this suggested that some MtrR::*glnE* DNA complexes may be weak or non-specific.

MtrR-binding sites upstream of *glnE* on both the sense and anti-sense strands were detected by DNase I protection. On the sense strand, we detected a relatively long sequence (82 nucleotides) that had regions protected by MtrR (Figure 3B). This region of protection encompassed two of the predicted MtrR binding sites and overlapped (16 nucleotides) the predicted MtrR-binding site that had the highest identity to previously identified binding sites [8,9,10] (Figure 2). Additionally, we detected DNase I hypersensitive regions on the sense strand, which were near the annotated *glnE* promoter. While no clear areas of protection were evident at these DNase I hypersensitive sites, their presence suggested that DNA-protein interactions occurred in or near these regions and resulted in alterations in the local DNA structure. On the anti-sense strand, we observed two protected sites (labeled as A′ and B′ in Figure 2 and Figure 3B), one of which straddles the annotated *glnE* promoter (site A′), while the other was located further downstream (site B′). Site A′ is notable as it overlaps the 27 bp region that was identified as being a possible MtrR-binding site. Of interest, we also noted a DNase I hypersensitive C residue in site A′ (see asterisk in Figure 2 and Figure 3B) that became apparent in the presence of MtrR; this site overlaps the annotated promoter region on the complementary strand.

Examination of the nucleotide sequence surrounding this DNase I hypersensitive C nucleotide revealed the presence of an imperfect seven nucleotide indirect repeat element (TAGCTTC/CAACGAT) that intervenes the annotated −35 and −10 hexamers. Examination of the MtrR-protected region identified two interesting features regarding the putative *glnE* promoter region. First, we noted the presence of a potential extended −10 motif (TGATATAGT) [25], previously observed for other −10 sequences in *N. gonorrhoeae* [26] that could mitigate the impact of a poor −35 element that was identified in the annotated sequence. Second, we identified an alternative −35 hexamer sequence (TTCAGA) that is more consistent with the sigma-70 consensus −35 hexamer sequence (TTGACA) in *E. coli*. This alternative −35 sequence would change the spacing between the annotated −10 hexamer from an optimal 17 nucleotides to a suboptimal 21 nucleotides or 18 nucleotides from the proposed extended −10 element. For either −10 element, the binding of MtrR to this region could enhance interaction of the promoter with RNA polymerase leading to increased transcription of *glnE*. In this respect, it is of interest that BmrR of *Bacillus subtilis* activates transcription of the *bmr*-encoded efflux transporter gene by binding to an imperfect inverted repeat element within the respective promoter that also has sub-optimal spacing (19 nucleotides) between the −10 and −35 hexamers [1,2].

In the case of MtrR activation of *glnE*, we do not yet know if all of the binding sites identified by DNase I protection are important in such regulation. However, given the gene activation model for BmrR [1], as a model to help explain the data we hypothesize that binding Site A′, which contains the imperfect indirect repeat element (Figure 2) between the −35 and −10 domains, is important in *glnE* activation by MtrR. Moreover, since the −10 element of the *glnE* promoter is similar to other extended −10 element bearing promoters in gonococci [26] and *E*. *coli* [25], the ability of MtrR to bind to this region could facilitate interactions of RNA polymerase with the promoter.

Our past and current studies on MtrR control of gonococcal genes, especially its opposing action on *glnA* and *glnE* (Figure 1), emphasize that this member of the TetR family of DNA-binding proteins, which are known [2] for their ability to transcriptionally repress efflux pump-encoding genes, can also function as a repressor or activator of “off-target” genes. Is there data to believe that the ability of MtrR to repress its main target, the *mtrCDE* efflux pump operon, as well as its capacity to activate expression of “off target” genes (e.g., *glnE*) is important *in vivo*? Based on the findings of Warner *et al.* [17,18] that employed an experimental murine model of lower genital tract infection in females, we posit that the answer is yes. In this infection model, which measured fitness during a dual infection by MtrR-positive and MtrR-negative isogenic gonococci, loss of MtrR was found to enhance fitness of gonococci during the first 4–5 days, but this advantage waned at later time points. The early competitive advantage due to loss of MtrR was linked to enhanced expression of the *mtrCDE*-encoded efflux pump, which exports host-derived antimicrobials such as antimicrobial peptides [27] and progesterone [18]. As the infection progressed, the observed diminution of the competitive advantage afforded by loss of MtrR was suggested to be due to the inability of gonococci to activate certain genes. Based on this proposal, we hypothesize that MtrR activation of *glnE* expression could contribute to maximal gonococcal fitness and growth during late stages of infection at mucosal surfaces where levels of glutamine are often low [22]. This hypothesis is consistent with the notion that glutamine synthesis is important for virulence in other bacterial pathogens (e.g., *Salmonella typhimurium*) [22].

The results presented herein are, to our knowledge, the first report of a repressor of drug efflux genes that can also directly activate a gene involved in basic metabolism. Taken together, we suggest that our work highlights the diverse global regulatory properties that could be displayed by DNA-binding proteins, such as MtrR, known for their capacity to negatively regulate drug efflux genes. Such regulation of “off target” genes and the consequences for bacterial physiology should be considered in research studies dealing with transcriptional regulation of drug efflux pump-encoding genes and bacterial resistance to antimicrobials.

## 3. Experimental Section

### 3.1. Quantitative Real-Time Reverse Transcription-Polymerase Chain Reaction (qRT-PCR)

qRT-PCR was used to confirm the microarray data reported by Folster *et al.* [7] and was performed as described by Katzif *et al.* [28]; a portion of the RNA isolated from isogenic strains FA19 and JF1 used in [7] was employed (kindly provided by L. Jackson and D. Dyer (University of Oklahoma Health Sciences Center, Oklahoma City, OK, USA)). cDNA was synthesized in an reverse transcription (RT) reaction with random hexamer primers and Superscript II RT (Invitrogen, Carlsbad, CA, USA). Specific primers for *glnE* and 16S RNA (internal control) used in PCR were 5'-ACTTCCCGCCACAATTTCCT-3' plus 5'-CGACGAATTGCTGTCCCATT-3' (*glnE*) and 5'-CATCGGTATTCCTCCACATCTC-3' plus 5'-TAGGGTGCGAGCGTTAATC-3' (16S RNA). qRT-PCR was performed on an iCycler iQ real-time PCR detection system (Bio-Rad Laboratories, Hercules, CA, USA). iQSYBR Green Supermix (Bio-Rad Laboratories) was employed in a reaction volume of 25 µL with 200 nM of 5' and 3' primers and five-fold dilutions of RT reaction mixtures.

### 3.2. Electrophoretic Mobility Shift Assays (EMSA) and DNase I Protection

EMSA and DNase I protection were used to test MtrR binding to the DNA sequence upstream of *glnE*. The production and purification of the maltose-binding protein (MBP)-MtrR fusion protein used in this study has been described previously [10]. This fusion protein was used in electrophoretic mobility shift assays (EMSA) and DNase I protection studies using previously described methods [7,9,10]. A PCR product that encompassed the upstream region of *glnE* was generated from chromosomal DNA from strain FA19 [7] using oligonucleotide primers 5'pglnE (5'-CAAGTGAGGAGCTGCATGAA-3') and 3'pglnE (5'-CGGGACATTTCGGATTCCGTTTG-3'), which were end-labeled with [γ-^32^P] dATP using T4 polynucleotide kinase (New England Biolabs, Beverly, MA, USA) as described previously [7,10]. The labeled PCR generated product was incubated with purified MtrR-MBP in a final reaction volume of 30 μL consisting of the reaction buffer (10 mM Tris-HCl (pH 7.5), 0.5 mM dithiothreitol, 0.5 mM EDTA, 4% (*v/v* glycerol, 1 mM MgCl_2_, 50 mM NaCl, poly(dI-dC) (0.05 μg/mL)) and dH_2_O at 25 °C for 30 min. Following incubation, the reactions were subjected to gel electrophoresis utilizing a 6% (*w/v*) polyacrylamide gel at 4 °C, dried, and autoradiography was performed for visualization. Competitive EMSAs were performed in the same manner, but with the addition of unlabeled specific competitor, generated from the same sequence as the target, or non-specific competitor, generated from the *rmp* gene using primers rmpF (5'-ATGTTTCTACAGCGGCCTG-3') and rmpR (5'-CGGCAAGATATTACCTAGCCT-3'). DNase I protection assays were performed essentially as described previously [7,10]. PCR-generated target DNA sequences were synthesized using oligonucleotide primers and were labeled at the 5' end of one strand as described above for EMSA. Purified MtrR-MBP was then incubated with the target DNA sequence under the same binding conditions as used in the EMSA for 15 min at 37 °C. Following the addition of MgCl_2_ (5 mM) and CaCl_2_ (2.5 mM), DNase I (Promega, Madison, WI, USA) was added and the reactions were incubated at 37 °C for 1 min. The reactions were stopped with DNase I stop buffer (95% ethanol, 7.5 mM ammonium acetate, and nuclease free H_2_O), snap frozen on dry ice for 15 min, and then precipitated at −80 °C overnight. The pellet was then washed in 70% (*v/v*) ethanol, dried, and resuspended in gel loading buffer (Epicentre, Madison, WI, USA). Reactions were then loaded on 8% denaturing polyacrylamide gel, subjected to gel electrophoresis, dried, and autoradiography was performed for visualization as described previously [7,10].

## 4. Conclusions

MtrR is a multi-tasking transcriptional regulatory protein of *N.* *gonorrhoeae* that directly represses expression of the *mtrCDE*-encoded multidrug efflux pump operon and differentially modulates expression of genes (*glnA* and *glnE*) involved in glutamine biosynthesis. Along with its capacity to control expression of other “off target” genes, the global regulatory action of MtrR likely serves to fine-tune important physiological processes of gonococci during infection.
